# Our Book Table

**Published:** 1884-11

**Authors:** 


### OUR BOOK TABLE.

Manual and Dental Directory of the United States. M. P. Beecher, Editor and Compiler. Beecher & Co., Publishers, New York, 1884.

This is quite a pretentious book of 163 pages. The first part or "Manual" contains many statistics of dentistry derived from various sources (some of them apparently from Caulk's Manual), extracts from valuable papers and from dental journals, "hints" and "facts" that are not always facts. There are some valuable "recipes" and some that will sorely test the patience of the dentist who attempts to make use of them.

The second part professes to present a complete directory of the dentists of the United States. This, if reliable, would be of great value to almost every practicing dentist, while to many it would make the book absolutely essential. But if the lists be incorrect, the book is worthless, for it will only mislead where precision is necessary. We cannot speak concerning all the lists, but if they are no more reliable than those with which we are acquainted, but little dependence can be placed upon them. We find the names of those long dead, and of those who never were dentists, while the names of many who are in reputable practice are entirely omitted. There seems to have been a desire to present a long list, with little care to make it a correct one.

Visions of Fancy. *A Poetical work,* by N. M. Baskett, M. D., Moberly, Mo. St, Louis: J. H. Chambers & Co., 1884.

This is not a poetical work, but a volume of fugitive and disconnected poems, none of which are of a sustained character. Some of them give evidence of considerable poetic feeling, but the greater part, while correct in rhythm and possessed of a jingling rhyme, are commonplace. To those who have a personal acquaintance with the writer the book may be of interest, but the casual reader will scarcely become very much absorbed in it.

The Physician's Visiting List for 1885. P. Blakiston, Son & Co., Philadelphia.

This is the thirty-fourth year of publication of this capital manual. In addition to the visiting list for the year, it contains a vast amount of information within a small compass, that is essential to every physician. There are posological tables, and tables of weights and measures, poisons and their antidotes, equivalents, and many other things, for which the reader is referred to the book itself.

TRANSACTIONS OF THE ILLINOIS STATE DENTAL SOCIETY, 1884.

We are indebted to the Secretary, Dr. J. W. Wassall, of Chicago, for a copy of this book. The Illinois State Dental Society is one of the very best in existence. The papers read before it are almost invariably of permanent value, and the discussions are what might be expected when one knows of what a class of men the Society is composed. The volume for 1884 is quite equal to that of most previous years, and we could not well say more in its praise.

TRANSACTIONS OF THE DENTAL SOCIETY OF THE STATE OF NEW YORK.

The transactions of the Society have usually been so long delayed in their publication that all interest in them has been lost before their reception. Much credit is due the Secretary, Dr. J. Edward Line, that a new departure has been taken and the volume issued promptly. Some of the papers this year are of great value, but the discussions are not as full as they should have been. The volume makes a creditable appearance, as a whole.

